# MSH2 and CXCR4 involvement in malignant VIPoma

**DOI:** 10.1186/1477-7819-10-264

**Published:** 2012-12-11

**Authors:** Sven Müller, Susan Kupka, Ingmar Königsrainer, Hinnak Northoff, Karl Sotlar, Thomas Bock, Reinhard Kandolf, Frank Traub, Alfred Königsrainer, Derek Zieker

**Affiliations:** 1 Department of General, Visceral and Transplant Surgery, Tübingen, Germany; 2 Department of Transfusion Medicine, Tübingen, Germany; 3 Department of Molecular Pathology, Tübingen, Germany; 4 Comprehensive Cancer Center, University of Tübingen, Tübingen, Germany; 5 Department of General, Visceral and Transplant Surgery, Comprehensive Cancer Center, University of Tübingen, Hoppe-Seyler-Strasse 3, Tübingen, D 72076, Germany

**Keywords:** VIPoma, MSH2, CXCR4, Microarrays, LOH

## Abstract

**Background:**

Vasoactive intestinal polypeptide secreting tumors(VIPomas) are rare endocrine tumors of the pancreas with an estimated incidence of 0.1 per million per year. The molecular mechanisms that mediate development of VIPomas are poorly investigated and require definition.

**Methods:**

A genome- and gene expression analysis of specimens of a primary pancreatic VIPoma with hepatic metastases was performed. The primary tumor, the metastases, the corresponding healthy tissue of the liver, and the pancreas were compared with each other using oligonucleotide microarrays and loss of heterozygosity (LOH).

**Results:**

The results revealed multiple LOH events and several differentially expressed genes. Our finding of LOH and downregulation was conspicuous in the microarray analysis for the mismatch repair gene MSH2 in the primary pancreatic VIPoma tumor, the hepatic metastasis but not in the corresponding healthy tissue. Further a strong overexpression of the chemokine CXCR4 was detected in the hepatic metastases compared to its pancreatic primary. With a review of the literature we describe the molecular insights of metastatic development in VIPoma.

**Conclusion:**

In VIPoma, defects in the mismatch repair system especially in MSH2 may contribute to carcinogenesis, and increased CXCR4 may be associated with liver metastasis.

## Background

Vasoactive intestinal polypeptide secreting tumor (VIPoma) is a rare tumor associated with watery diarrhea, hypokalemia, and achlorhydria (WDHA) [1]. The estimated incidence is approximately 0.1 per million per year [2]. VIPomas are mostly located in the pancreas, although they have been already detected in the bronchial system, colon, liver, adrenal gland, and sympathetic ganglia [3,4]. The major causes of death concerning VIPomas are dehydration and renal failure [5]. Clinical symptoms lead to the diagnosis. When symptoms present several patients already have metastasis. The only curative approach is surgical resection [6]. Until now the molecular mechanisms that mediate development of metastases of VIPomas require definition and were not investigated yet. Thus, we present this rare entity by investigating a genome- and gene expression analysis of specimens of a primary pancreatic VIPoma with hepatic metastases.

## Methods

### Case presentation

**Figure 1:**
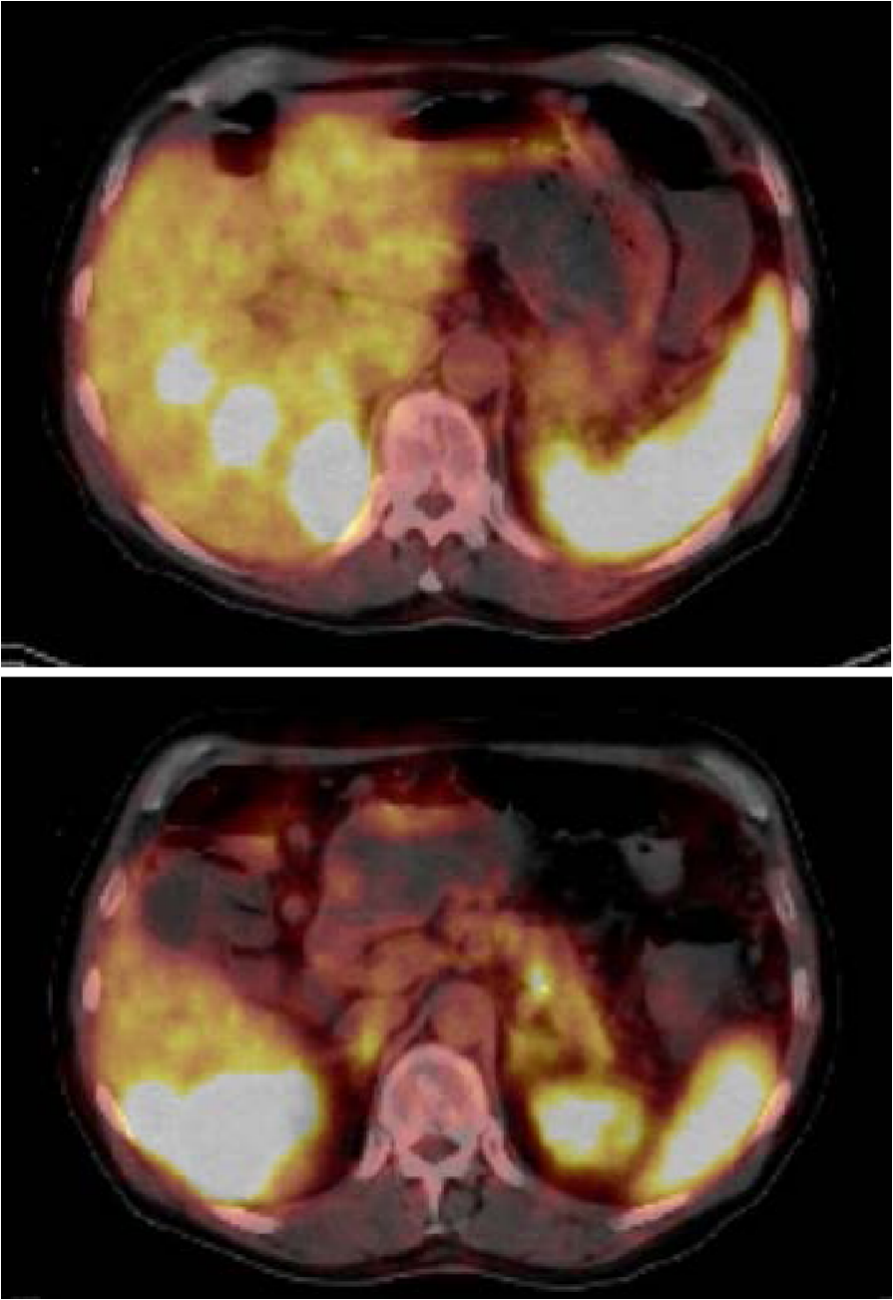
PET-CT with MBq 68-Gallium-DOTATOC showing enhancement of multiple lesions in the right lobe of the liver (upper image) and a slight enhancement in the caudal pancreas (lower image).

A 69-year-old man was referred from an outside hospital to our department with masses in the liver and therapy-resistent watery diarrhea. Since 1996 he had suffered from protracted watery diarrhea. At that time the patient received oral rehydration solutions and electrolyte supplementation without regression of the symptoms. In September 1999 ultrasonography was performed and several hepatic masses were detected. Subsequent biopsy of the hepatic masses revealed the diagnosis of metastases due to a malignant VIPoma without evidence of the location of the primary. For further conservative treatment the patient received Sandostatin without regression of the diarrhea. Thus, the patient was administered Rituximab and Roferon additionally, so the progress of the symptoms was impeded. During the course of treatment, minor relapses were compensated using Capecitabine and Cetuximab as additional medication. Regarding side effects of therapy,the patient developed enormous acne. In December 2005 the patient stopped responding to the performed conservative treatment and suffered from massive watery diarrhea (5 to 10 L/day), hypokalemia, achlorhydria, anemia, and severe metabolic acidosis with consecutive acute renal failure and requiring dialysis. Finally the patient was referred from an outside hospital and was admitted to our hospital and department in February 2006. At admission computed tomography (CT) and magnetic resonance imaging (MRI) were performed. In the liver a 4.7 × 3.3 cm mass in segment VII, a 6.6 cm mass in diameter in segment V/VI, and two lesions with 2 cm and 1.7 cm in diameter in segment V were detected. The findings showed an increase in size compared to a previous CT scan performed in July 2005. Since CT and MRI were not able to reveal the location of the primary a PET-CT with MBq 68-Gallium-DOTATOC was conducted. Enhancement of multiple lesions in the right lobe of the liver and a slight enhancement in the caudal pancreas without morphological circumscribable primary tumor were observed (see Figure 1). No infiltration of lymph nodes or other organs was detected. Due to the censorious condition of the patient, at first a tumor-debulking to decrease the amount of VIP causing the symptoms was suggested. Plasma levels of VIP were between 450 and 650 pmol. Accordingly a hemihepatectomy was conducted in March 2006 whereas the surgeon was able to palpate a lesion in the caudal pancreas intraoperatively. Based on the severe condition of the patient, a simultaneous hemihepatectomy and pancreasectomy was not able to be performed at that time without putting patient’s life at risk. A liver weighing 912 g and measuring 18.5 × 17.7 × 8.4 cm was resected. Pathologic examination and immunohistochemistry revealed metastases due to a malignant VIPoma and expression of Chromogranin-A and Synaptophysin. In time the condition of the patient stabilized. The symptoms improved and the plasma VIP decreased (18 pmol). After a reasonable recovery the patient underwent caudal pancreasectomy and splenectomy in January 2007 with successful resection of the primary tumor *in sano*. A pancreas specimen measuring 9 × 5 × 3 cm was resected. Pathologic examination and immunohistochemistry revealed a pancreatic neuroendocrine tumor confirming a malignant VIPoma with expression of Chromogranin-A and Synaptophysin. The postoperative course was uneventful and the patient did not show any further symptomatic. The follow-up examinations with CT and MRIdid not reveal any recurrence or clinical symptoms, and VIP plasma levels were low (2 pmol).

### Patients and tissue specimens

Histological proven tissue samples (primary pancreatic VIPoma with hepatic metastases and corresponding healthy tissue of the liver and the pancreas) of this patient were investigated. All tumor specimens were collected by the Department of General, Visceral and Transplant Surgery, University of Tübingen, Germany. Specimens were snap frozen in liquid nitrogen and stored at −80°C until use. Each tumor sample was cryosected, stained with hematoxylin and eosin, classified by two experience pathologists, and re-evaluated by an experienced surgical pathologist. To ensure a tumor content >80%, microdissection was performed regarding cancer specimens. The patients provided signed informed consent. The study was approved by the local Ethics Committee.

### RNA isolation

RNA was extracted using the NucleoSpin RNA II Kit (Macherey-Nagel, Dueren, Germany). The RNA quality and quantity were verified using the Agilent 2100 Bioanalyzer (Agilent Technologies, Palo Alto, CA, USA) and the NanoDrop ND-1000 spectrophotometer (NanoDrop Technologies, Wilmington, DE, USA).

### Microarray data generation and statistical analysis

Material and Methods concerning production and analysis of our oligonucleotide microarrays have been described already by Zieker *et al*. [7,8]. In short:

Microarray analysis was performed using oligonucleotide microarrays (65mer) produced at the Max Planck Institute, Tübingen, Germany. The arrays contained oligonucleotides for about 900 transcripts, and each oligonucleotide was printed twice. Using 5 oligonucleotide microarrays (including dye-swap) we compared the primary tumor, the metastases, the corresponding healthy tissue of the liver, and the pancreas. Amplification of the sample RNA was performed using Ambion’s Amino AllylMessageAmp™ II aRNA Amplification Kit (Ambion Inc., Austin, TX, USA). Dye-coupling reaction was performed using Amersham CyDye Post-labelling Reactive Dye Pack (GE Healthcare, Buckinghamshire, UK). After an aRNA fragmentation using Ambion’s Fragmentation Reagents (Ambion Inc., Austin, TX, USA), hybridization was carried out at 48°C for 14 h. The slides were scanned in a microarray scanner (Genetix Limited, Hampshire, UK). The photomultiplier tube voltage was set to 100% for both red and green channels. The two resulting green and red images were overlaid using ImaGene 5 (BioDiscovery, Inc., El Segundo, CA, USA). Raw data collection was performed using ImaGene v.5.0. Further statistical and bioinformatic analyses were performed using the R language (http://www.r-project.org) and the ‘limma’ (Linear Models for Microarray) package from the Bioconductor project (http://www.bioconductor.org/). As a first step in signal extraction, for each channel we used the mean of the pixel distribution for the foreground signal and the median for the background of each spot as estimators of the raw signal values. All spots were used, regardless of their flag status. The data were normalized using loess normalization on the normexp-background corrected expression values, followed by a dye-swap normalization and in-between-array quantile normalization. Both the loess and quantile normalization methods were used as provided in the limma package. On the basis of the M values computed, differentially expressed genes were detected by applying the Welch one-sample *t*-test as implemented in R.

### Microsatellite analysis

DNA was extracted from both tissue and patients’ blood using the Gentra-DNA-extraction kit (Biozym) according to the manufacturer’s instructions. The following DNA samples derived from patients’ material were used for microsatellite analysis:

M32: primary tumor

M32I: liver metastasis 1

M32II: liver metastasis 2

M32N: normal liver tissue

Data derived from these samples were compared to results derived from blood DNA.

### LOH analysis

Microsatellite analysis was performed applying an extended marker panel for endocrine tumors which was previously established in our department [9]. In brief, markers BAT25, BAT26, D2S123, and D17S250 were amplified in a multiplex PCR (multiplex 1) using the QIAGEN Multiplex PCR Master Mix (Qiagen) under following conditions: final concentration of each forward primer (dye labeled) was 0.2 μM, of each reverse primer 1 μM, DNA concentration was 100 ng. Asymmetric PCR was used to reduce the ratio between primer signal and amplicon signal thereby improving the fluorescence detection threshold. Thermal cycling conditions were 94°C for 5 min (94°C for 30 s, 54°C for 90 s, 72°C for 60 s), 30 cycles, 60°C for 5 min. PCR products were purified using the QIAquick PCR purification kit (QIAGEN).

Microsatellite markers D2S443, D16S752, D21S1436 (multiplex 2) and D1S104, D3S1284 (multiplex 3) were amplified in two multiplex PCR reactions under following conditions: 200 ng DNA, 1 × PCR buffer, 0.08 mM dNTPs each, 2 mM MgCl_2_, 1 U Taq, 0.8 μM primer each, ad 50 μL H2O; 94°C for 2 min (94°C for 40 s, 53°C (multiplex 2), and 61°C (multiplex 3) for 40 s, respectively, 72°C for 1.30 min), 40 cycles, 72°C for 2 min. PCR products derived from the same patients sample were combined and purified using the PCR-purification kit (Qiagen). LOH analysis was performed on an automated capillary sequencer CEQ 8000 (Beckman Coulter) and evaluated with the CEQ 8000 Fragment analysis software (Beckman Coulter).

### LOHscoring

The tumor was considered to be positive for LOH if the allele peak ratio was ≤0.7, in analogy with allelic signal reduction of at least 30% [10]. Homozygous peaks were classified as not informative and were not evaluated in LOH statistics.

### Literature review

A Medline search was conducted for the term ‘VIPoma’to December 2011. A total of 489 publications were retrieved. All English written publications reporting on oncogenesis were included. Articles dealing with clinical findings, diagnostics, therapy options, and clinical outcomes were excluded.

## Results

### Microarray analysis

Gene expression analysis was performed using a custom-designed oligo microarray. We found a number of genes that were up- or downregulated comparing the primary tumor, the metastases, the corresponding healthy tissue of the liver, and the pancreas.

Concerning the expression ratio we found 36 genes to be differentially expressed (>log2). Eighteen genes upregulated and 18 genes were downregulated. Comparing the primary pancreatic tumor against its corresponding healthy tissue six genes were upregulated and eight genes were downregulated (>log2) (See Table 1). Five genes were upregulated and five genes were downregulated comparing the primary pancreatic tumor with the hepatic metastases (>log2) (see Table 2). Regarding the expression between the hepatic metastases and its corresponding healthy liver tissue, seven genes were upregulated and five genes were downregulated (>log2) (see Table 3).

**Table 1:** Comparison of gene up- and downregulation of the primary pancreatic tumor against its corresponding healthy pancreatic tissue (>log2)

**Geneproduct upregulated**	**Log2ratio**	**Geneproduct downregulated**	**Log2ratio**
Homo sapiens SMAD family member 5 (SMAD5)	2.60	Homo sapiens catenin (CTNNAL1)	−2.01
Homo sapiens neural cell adhesion molecule 2 (NCAM2)	2.56	Homo sapiens adhesion regulating molecule 1 (ADRM1)	−2.04
Homo sapiens cadherin-like 26 (CDH26)	2.46	Homo sapiens histonedeacetylase 2 (HDAC2)	−2.29
Homo sapiens S100 calcium binding protein A9 (S100A9)	2.32	Homo sapiens cyclin L2 (CCNL2)	−2.34
Homo sapiens mediator of DNA damage checkpoint 1 (MDC1)	2.25	Homo sapiens MSH2 (MSH2)	−2.63
Homo sapiens adhesion molecule, (AMICA1)	2.24	Homo sapiens SMAD family member 6 (SMAD6)	−2.71
		Homo sapiens guaninenucleotide binding protein (G protein)	−2.72
		Homo sapiens cyclin-dependent kinase 5, (CDK5R2)	−3.02

**Table 2:** Comparison of gene up- and downregulation of the primary pancreatic tumor against the hepatic metastases (>log2)

**Geneproduct upregulated**	**Log2ratio**	**Geneproduct downregulated**	**Log2ratio**
Homo sapiens cyclin-dependent kinase 7 (CDK7)	4.52	Homo sapiens acyl-CoA synthetase, (ACSM1)	−2.17
Homo sapiens cadherin 4 (CDH4)	3.72	Homo sapiens cadherin 20, type 2 (CDH20)	−2.27
Homo sapiens cyclin D2 (CCND2)	3.06	Homo sapiens HUS1 checkpoint (HUS1B)	−2.67
Homo sapiens gapjunctionprotein, beta 5 (connexin 31.1) (GJB5)	2.73	Homo sapiens chemokine (C-X-C motif) receptor 4 (CXCR4)	−3.37
Homo sapiens lipase, hormone-sensitive	2.25	Homo sapiens caudal type homeobox ranscription factor 2 (CDX2)	−3.39

**Table 3:** Comparison of gene up- and downregulation of the hepatic metastases and its corresponding healthy liver tissue (>log2)

**Geneproduct upregulated**	**Log2ratio**	**Geneproduct downregulated**	**Log2ratio**
Homo sapiens neuronal cell adhesion molecule (NRCAM)	2.76	Homo sapiens guanine nucleotide binding protein-like 2 (GNL2)	−2.23
Homo sapiens GNAS complex locus (GNAS)	2.58	Homo sapiens alanine-glyoxylate aminotransferase homolog (TLH6)	−2.38
Homo sapiens cell division cycle and apoptosis regulator 1 (CCAR1)	2.51	Homo sapiens MSH2 (MSH2)	−2.80
Homo sapiens cyclin-dependent kinase inhibitor (CDKN1C)	2.48	Homo sapiens gap junction protein, beta 1 (GJB1)	−2.87
Homo sapiens cytochrome c oxidase subunit VIIa polypeptide 2 (liver)	2.29	Homo sapiens caspase 1 (CASP1)	−3.21
Homo sapiens mucin and cadherin-like (MUCDHL)	2.09		
Homo sapiens cyclin D1 (CCND1)	2.04		

Regarding the gene expression of MSH2 and CXCR4 we were able to detect a downregulation (−2.80-fold) of MSH2 in hepatic metastases and (−2.63-fold) of MSH2 in the primary pancreatic tumor compared to both corresponding healthy tissue. Comparing the hepatic metastases against the primary pancreatic tumor an upregulation (10.41-fold) was detected.

### LOH results

Ten microsatellite samples were used for MSI and LOH detection (see Table 4).

**Table 4:** Microsatellite markers used for MSI and LOH detection in pancreatic tumor tissue, liver metastasis tissue, and normal liver tissue

		**Tissue**	**Pancreatic tumor**		**Liver metastasis**		**Normal liver**	
*Marker*	*Localization*			AP ratio		AP ratio		AP ratio
D1S104	1q21-23		LOH	0.2	LOH	0.49	MSS	>0.7
D2S443	2p13		LOH	0.19	LOH	0.42	MSS	>0.7
BAT26	2p16		MSS	>0.7	MSS	>0.7	MSS	>0.7
D2S123	2p16		LOH	0.21	LOH	0.45	MSS	>0.7
D3S1284	3p12		LOH	0.19	LOH	0.47	MSS	>0.7
BAT25	4q12		MSS	>0.7	MSS	>0.7	MSS	>0.7
D5S326	5q22.2		LOH	0.2	LOH	0.31	LOH	0.32
D16S752	16q22		LOH	0.12	LOH	0.39	MSS	>0.7
D17S250	17q11.2-12		MSS	>0.7	MSS	>0.7	MSS	>0.7
D21S1436	21q21		LOH	0.6	LOH	0.4	LOH	0.59

AP ratio, Allele peak ratio; LOH, Loss of heterozygosity; MSS, Microsatellite stable.

**Figure 2:**
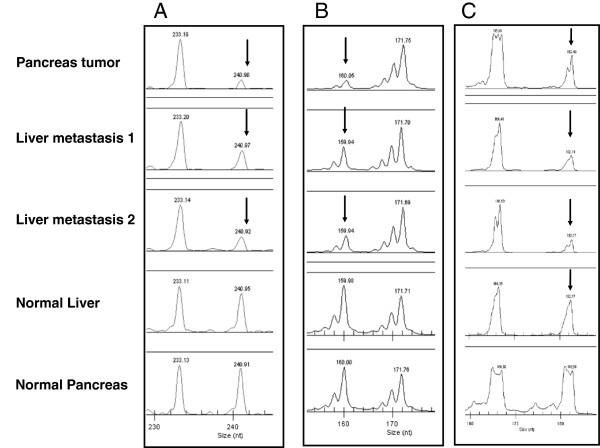
Loss of heterocygosity (arrow) for the markes D2S443 (A), D3S1284 (B), and D21S1436 (C) in pancreas tumor tissue, liver metastases tissue (1&2), normal liver and normal pancreas tissue.

All experiments were repeated at least twice. LOH events were detected in several patient samples (see Figure 2). Altogether, allele peak ratios for LOH calculation varied between 0.60 and 0.12.

Sixty-three percent of markers analyzed showed LOH. Nine percent of markers were not informative for LOH calculation and 27% of markers showed microsatellite stability. LOH was detected in primary tumor DNA as well as in liver metastasis. Interestingly, two markers (D5S326 and D21S1436) revealed LOH in normal liver tissue. All tumor DNAs analyzed showed LOH for marker D2S123 which is located close to the MSH2.

## Discussion

VIP-producing tumors arise from pancreatic islet cells and are mostly located in the pancreatic body or tail [4]. Malignancy is solely defined by existing distant metastasis [11]. The clinical symptoms accompanying the tumor are watery diarrhea, hypokalemia, and metabolic acidosis. At the time of presentation, over 70% of patients had hepatic metastases [12]. Hence, understanding the molecular mechanisms of tumor progression is of paramount importance. Our results revealed defects in the mismatch repair system especially in MSH2. MSH2 showed significantly lower expression in the primary pancreatic tumor and its liver metastases than in healthy pancreatic tissue. Further an overexpression of CXCR4 in the hepatic metastasis compared with the primary was detected.

Reviewing the literature,eight cohorts including 161 patients and 133 case reports exist on VIPoma. Only six publications deal with the molecular mechanisms of oncogenesis and metastasis development in VIPoma, reporting no more than two cases in each publication [13-18]. The remaining publication reports on the clinical presentation, diagnostics, and therapeutic options. Amongst others, mutations in the tumor suppression genes DPC4 [15], AIM1 (‘absent in melanoma1’) and PTPRK (receptor type protein-tyrosine phophastase kappa) [14] are shown in metastatic VIPomas while mutations of the proto oncogene BRAF known to play an important role in thyroid cancer and melanoma were not found [15,18]. A series of 35 neuroendocrine tumors of the pancreas showed that defects in DNA mismatch repair are rather rare with the limitation, that no VIPoma case was included [19].

The DNA mismatch repair system (MMR) corrects base mismatches after DNA replication, avoids recombination between non-identical DNA sequences, and induces apoptotic and checkpoint responses after DNA damage [20]. Besidespost-replication repair, MMR proteins are assumed to a great extent to be involved in carcinogenesis [21]. So far hundreds of different predisposing mutations are known, mainly affecting the MMR genes, MLH1, MSH2, and MSH6. Predisposed individuals exhibit a deficient copy of a MMR gene in each cell. Deficient MMR, characteristic in the colon can lead to proceeding accumulation of mutations and cancer, such as hereditary non-polyposis colon cancer (HNPCC), is known to be a syndrome of insufficient DNA mismatch repair genes [21]. Instability at short tandem repeat sequences, microsatellites, is a representative manifestation of MMR deficiency and beside HNPCC also occurs in other tumors [20]. In particular deficient MSH2 is associated with an increased risk of cancer [22].

Chemokines and their respective receptors have been identified as contributing metastatic factors in numerous cancers [23,24]. The common human chemokine system includes about 50 ligands and 20 G protein-coupled receptors which control migration and activation of leukocytes and influences angiogenesis and tumor growth [25]. Recent discoveries assume that tumor cells themselves are able to secrete chemokines [25-27]. Therefore disseminated tumor cells that express members of the CXCR family and invade the circulation may be attracted and arrested by their corresponding ligand. Hence these cells gain the ability to infiltrate in distinct organs. CXCR4 is assumed to be involved in metastasis of non-small-cell lung, breast, pancreatic, prostate, gastric cancer, and peritoneal carcinomatosis [27-32]. Deschamps *et al*. found CXCR4 to be associated with the malignant metastatic progression of neuroendocrine tumors of the ileum correlated with a lower survival [33]. So far, mutation of MSH2 and CXCR4 and its involvement in tumor growth and metastasis development have not been reported in VIPoma.

## Conclusions

Altogether, the altered expression of MSH2 and CXCR4 in a metastatic VIPoma might reflect higher tumor aggressivity with the potential development of distant metastases. Owing to the rarity of this disease, we believe these results will provide a valuable resource for future work on this serious condition with the potential of diagnostic applications.

## Competing interests

This manuscript contains original material that has not been published or submitted to any other journal. The authors declare no competing interests.

## Authors’ contributions

SM and DZ designed the study, analysed the data and drafted the manuscript. SK carried out the LOH analysis. IK and FT carried out the molecular studies and helped to draft the manuscript. TB, KS and RK provided the pathologic specimen and helped to draft the manuscript. HN and AK helped to draft the manuscript. All authors read and approved the final manuscript.
